# Bone metastasis from malignant phyllodes breast tumor: report of two cases

**DOI:** 10.1186/s12907-016-0027-7

**Published:** 2016-02-29

**Authors:** Mohamed Reda El Ochi, Mehdi Toreis, Mohamed Benchekroun, Zineb Benkerroum, Mohamed Allaoui, Mohamed Ichou, Basma El Khannoussi, Abderrahman Albouzidi, Mohamed Oukabli

**Affiliations:** Department of Pathology, Mohamed V military Hospital, Hay Riad, Faculty of Medicine, Mohamed V University, BP10000 Rabat, Morocco; Department of Medical Oncology, Mohamed V military Hospital, Hay Riad, Faculty of Medicine, Mohamed V University, Rabat, Morocco; Department of of Orthopaedics and Traumatology, Mohamed V military Hospital, Hay Riad, Faculty of Medicine, Mohamed V University, Rabat, Morocco; Department of of Gynecology and obstetrics, Mohamed V military Hospital, Hay Riad, Faculty of Medicine, Mohamed V University, Rabat, Morocco; Department of Pathology, National Institute of Oncology, Hay Riad, Faculty of Medicine, Mohamed V University, Rabat, Morocco

**Keywords:** Bone, Metastatic, Phyllodes, Tumor, Breast

## Abstract

**Background:**

Phyllodes tumors are rare fibroepithelial tumors accounting for less than 1 % of all breast neoplasms. They are malignant in 20 % of cases. Only a few cases of malignant phyllodes tumors metastatic to bone have been reported.

**Case presentation:**

Case 1: A 40 year-old white woman presented with three-week history of pain and functional impairment of the left lower limb. Her clinical past was remarkable for previous left mastectomy and radiotherapy for malignant phyllodes tumor performed one year ago. Computed tomography revealed a moth-eaten appearance of the left femoral head. The patient underwent computed guided femoral head biopsy. Pathological findings were consistent with metastatic malignant phyllodes tumor. The patient received ifosfamide and adriamycin chemotherapy. She is doing well without any evidence of progression on her imaging follow- up after 8 months.

Case 2: A 48 year-old white woman, with history of bilateral mastectomy and radiotherapy for malignant phyllodes tumor performed one and two year ago, presented with four-week left lower quadrant abdominal pain. Computed tomography and magnetic resonance imaging revealed a solid aggressive osteolytic mass of the left iliac bone with extensive soft tissue invasion. Biopsy of the tumor was performed and showed a sarcomatous proliferation consistent with metastatic malignant phyllodes tumor. The patient received the same chemotherapy regimen as in the first case but without any response on her imaging follow up after 6 months.

**Conclusion:**

Malignant phyllodes tumor is a rare and aggressive fibroepithelial neoplasm. An accurate diagnosis of metastases should be based on clinicopathological correlation allowing exclusion of differential diagnoses. The goal of successful managing this tumor is early detection and complete resection prior to dissemination.

## Background

Phyllodes tumors (PTs) are rare fibroepithelial tumors accounting for less than 1 % of all breast neoplasms [1, 2]. They are classified as benign, borderline and malignant [3].

Malignant PTs account for 20 % of all PTs [4] and may present with delayed metastases mainly in the lung [5].

Only a few cases of PT metastatic to bone have been reported [6]. To our knowledge, only 2 cases involving the iliac bone [6, 7] and 1 case involving the femur [8] are described in the literature. These papers have mainly focused on their radiological aspects. We report 2 cases of metastatic malignant PT of the breast involving the femoral head and the iliac bone and discuss the histopathological differential diagnoses.

## Case presentation

### Case 1: Clinical history

**Fig. 1:**
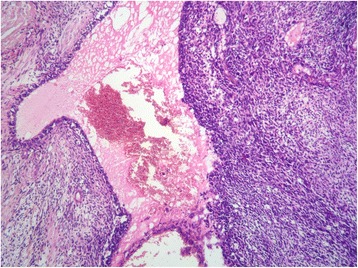
Malignant PT of the breast showing a leaf-like pattern with increased stromal cellularity and atypia (hematoxylin and eosin stain, original magnification × 100)

A 40 year-old white woman presented with a three-week history of pain and functional impairment of the left lower limb. Clinical examination showed limitation of the left lower limb movements. Her clinical past was remarkable for previous left mastectomy and for malignant PT performed one year ago. The mass measured 7x5x4 cm. Histological examination showed a biphasic proliferation characterized by a double layered epithelial component arranged in clefts surrounded by an hpercellular fibrosarcomatous component organized in leaf-like structures Fig. 1. There was stromal overgrowth, marked nuclear atypia and high mitotic activity (12 per 10 high-power fields). Surgical margins were complete and of at least 1 cm. Immunoreactivity with anti vimentin was found. Smooth muscle actin, desmin, CD34, S-100 protein, CD10, CKAE1/AE3, P63, estrogen and progesterone receptors were negative. Post operatively, the patient received radiation to the tumor bed at a dose of 50 Grays in 25 fractions without chemotherapy.

### Radiologic and histopathologic findings

**Fig. 2:**
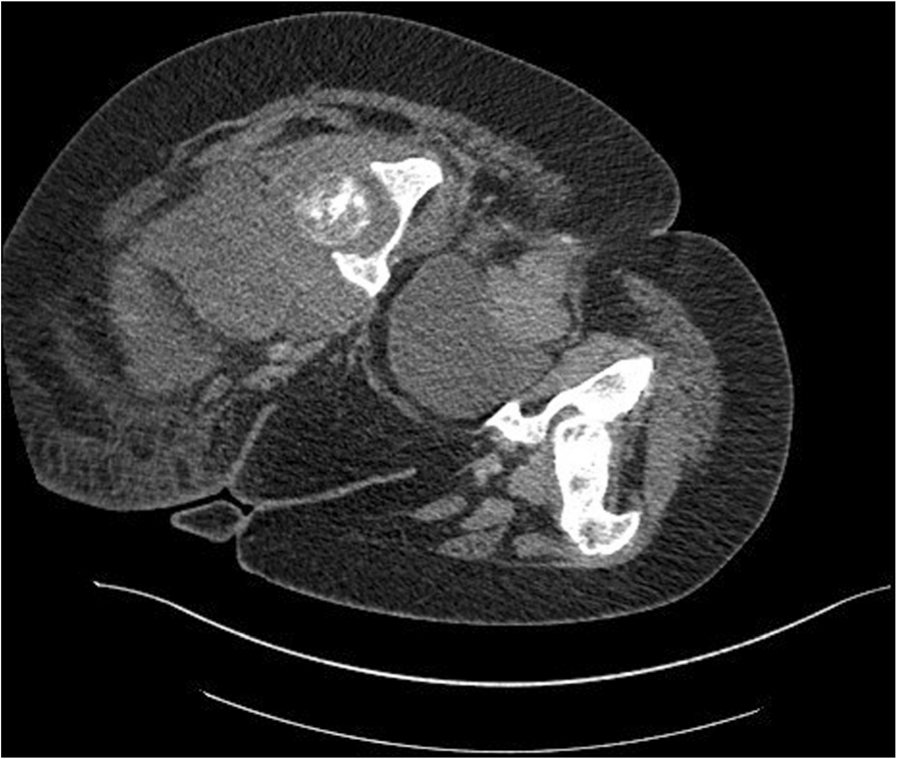
Axial computed tomography image showing a moth-eaten appearance of the femoral head

**Fig. 3:**
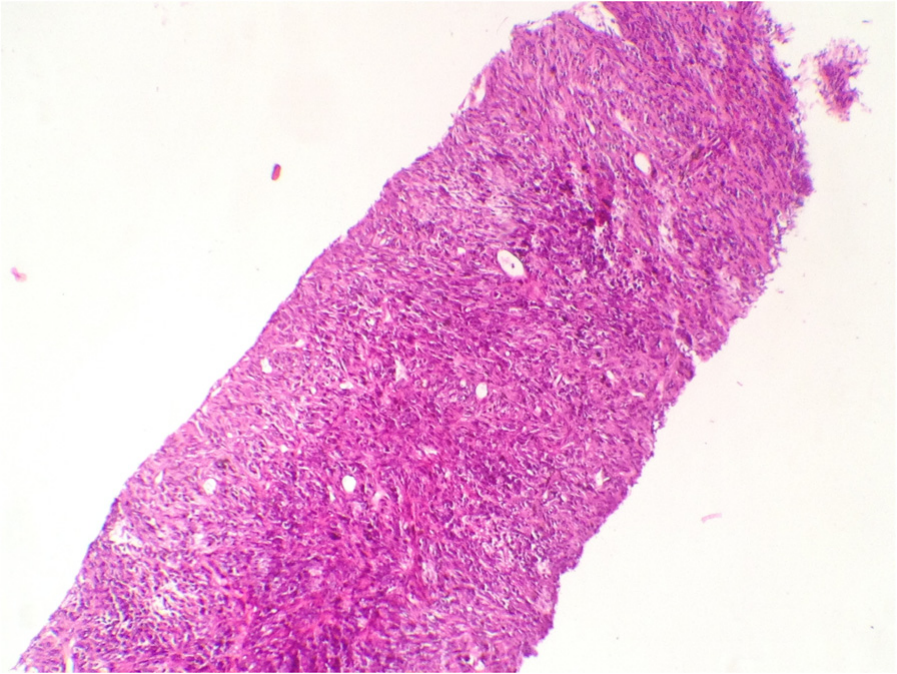
Fascicular proliferation of spindle shaped cells (hematoxylin and eosin stain, original magnification × 100)

**Fig. 4:**
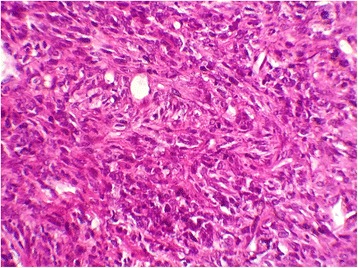
Tumor cells showing mild nuclear atypia (hematoxylin and eosin stain, original magnification × 400)

Computed tomography and FDG-PET Scan revealed a moth-eaten appearance Fig. 2 and pathological FDG uptake of the left femoral head without other suspect lesions. The patient underwent computed guided femoral head biopsy. The post-operative course was uneventful. Microscopic examination showed a proliferation of fascicles of spindle cells with nuclear atypia and numerous mitotic figures Figs. 3 and 4. No areas of epithelial, osteoid or chondroid components were identified. Immunohistochemistry demonstrated only vimentin positivity. Pancytokeratin, smooth muscle actin, desmin, S100 protein, CD34, CD31, CD99, CD117, estrogen and progesterone receptors were all negative. Thus, a diagnosis of metastatic

malignant PT was made. The patient received ifosfamide and adriamycin chemotherapy. She is doing well without any evidence of progression on her imaging follow-up after 8 months.

### Case 2: Clinical history

**Fig. 5:**
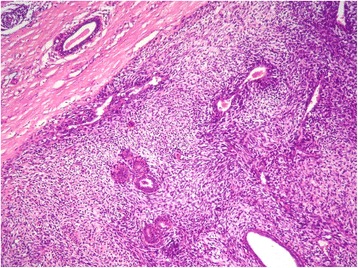
Malignant PT of the breast showing a periductal stromal growth with malignant features (hematoxylin and eosin stain, original magnification × 100)

A 48 year-old white woman presented with a four-week history of left lower quadrant abdominal pain. The patient’s past medical history was significant for previous bilateral mastectomy for malignant PT one and two year ago Fig. 5. The tumors measured 10x8x5 cm in the right breast (first mastectomy) and 6×5×4,5 cm in the left breast. They showed stromal overgrowth, marked nuclear atypia and high mitotic activity (greater than 14 per 10 high-power fields). Surgical margins were complete and of at least 0,7 cm on the right breast and 1,2 cm on the left breast. Immunoreactivity was found only with vimentin and CD10. The patient received radiation only to the right tumor bed at a dose of 50 Grays in 25 fractions without chemotherapy.

### Radiologic and histopathologic findings

**Fig. 6:**
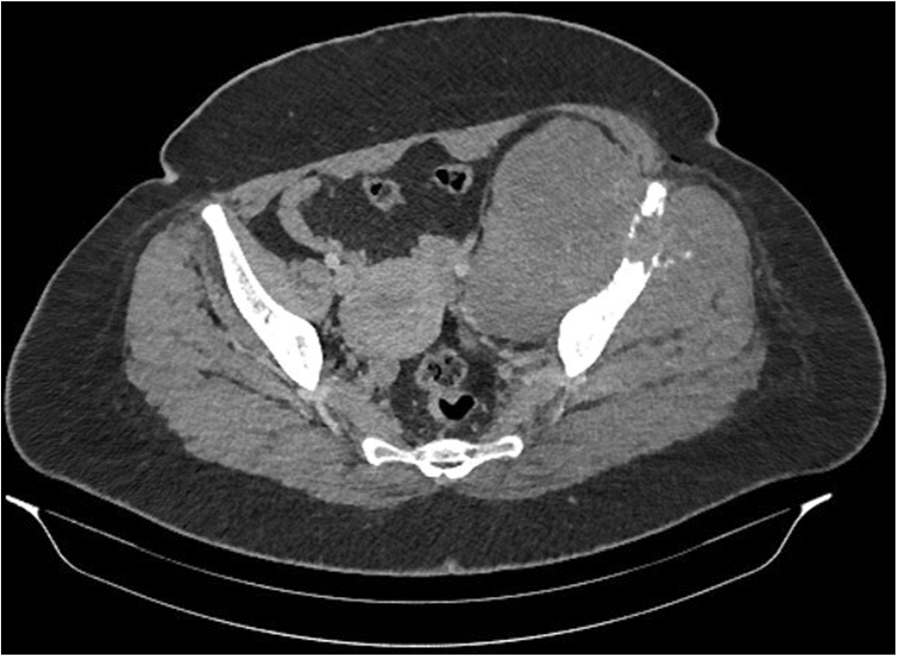
Axial computed tomography showing a solid aggressive osteolytic mass of the left iliac bone with extensive soft tissue invasion

**Fig. 7:**
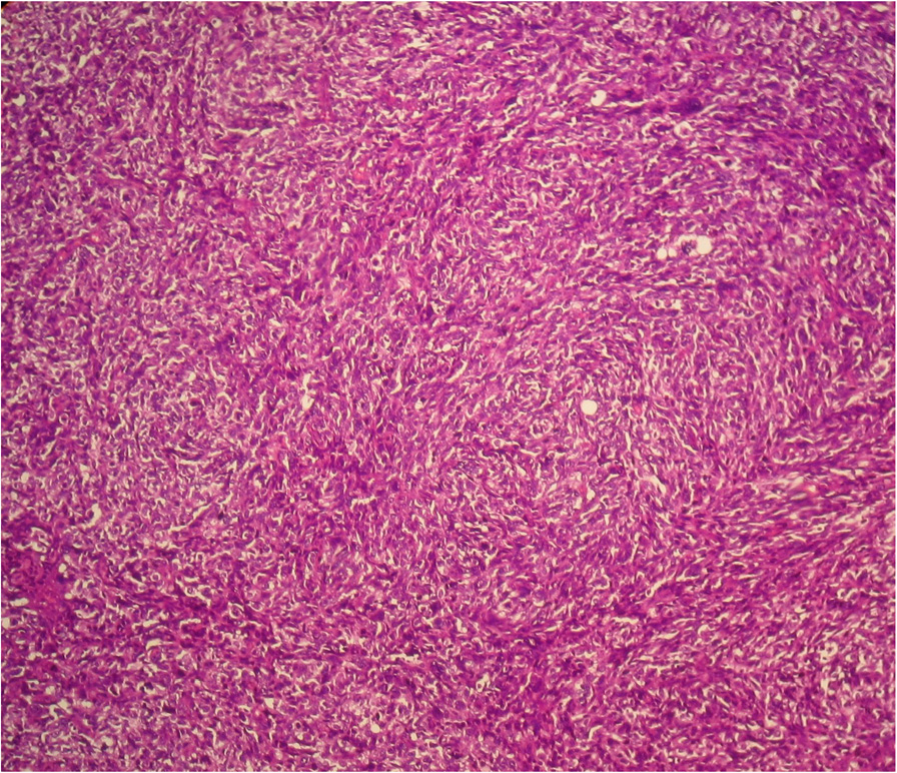
Proliferation of densely packed spindle cells (hematoxylin and eosin stain, original magnification × 100)

**Fig. 8:**
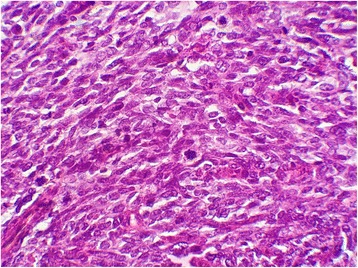
Tumor cells showing severe nuclear atypia with mitoses (hematoxylin and eosin stain, original magnification × 400)

Computed tomography and magnetic resonance imaging revealed a solid aggressive osteolytic mass of the left iliac bone with extensive soft tissue invasion measuring 13 × 11 cm Fig. 6. Biopsy of the tumor was performed and showed a sarcomatous proliferation similar to that described for the first case Figs. 7 and 8 with the same immunohistochemical profile. A diagnosis of metastatic malignant PT of the breast was made. The patient received the same chemotherapy regimen as in the first case but without any response after 7 months. The oncologist decided to use taxanes as second line chemotherapy with radiological stabilization on her imaging follow up.

## Discussion

PT is a rare fibroepithelial tumor accounting for less than 1 % of all breast neoplasms [1, 2]. They usually arise in women between ages 35 and 55 years and are classified as benign, borderline and malignant [2, 3]. Malignant types present approximately 20 % of all cases [4]. Actually, malignant PTs should be treated by conservative surgery with adequate negative surgical margins; the use of radiotherapy may be limited to patients with positive surgical margins [9, 10]. Distant metastases are seen in 10–20 % of cases [1]. They can occur even after technically adequate initial breast surgery [1]. The most reliable predictive factors for development of distant metastases are stromal overgrowth, nuclear pleomorphism and high mitotic activity [9, 11], whereas the role of tumor size and local recurrence is controversial [1, 11, 12]. Tan et al. found, by multivariate analysis, that stromal atypia, overgrowth, surgical margins and mitoses are independently predictive of clinical behaviour [13]. He developed a nomogram based on these criteria to predict recurrence-free survival, but the amalgamation of local with distant recurrences and the low rate of metastasis in this series could limit its ability in predicting dissemination. The recurrence free survival was 0,8 and 0,47 at 1 and 3 years for the first case, 0,76 and 0,4 at 1 and 3 years for the second case. Al-Masry et al. have shown that the expression of CD10 can be used to predict the occurrence of distant metastasis [14].

Metastatic PTs mainly develop from 3 to 10 years after the inital therapy, but they can be delayed or occur as soon as synchronous presentation [11]. The lung is the most common site of metastatic spread [2, 3, 15]. Only a few cases of PT metastatic to bone have been reported [6] with 2 cases involving the iliac bone [6, 7] and 1 case involving the femur [8].

Clinical features are not specific and vary among location of bone metastasis. Radiographs and computed tomography may show a solid mass adjacent to the involved bone and infiltrating the cortex and medulla in a permeative pattern [8]. The magnetic resonance imaging may better delineate the metastatic extent [8].

Pathological examination shows a malignant proliferation of fascicles of spindle cells with nuclear atypia and high mitotic index without epithelial component [15, 16].

Immunohistochemistry demonstrates only vimentin positivity. Pancytokeratin, smooth muscle actin, desmin, S100 protein, CD34, C31, CD99 and CD117 are generally negative [15, 16].

Positivity of estrogen and progesterone receptors had never been reported. These morphological and immunohistochemical findings play an important role in excluding sarcomas, myoepithelioma, metastatic sarcomatoid carcinoma, melanoma and gastrointestinal stromal tumor. Generally, it’s difficult to make a specific diagnosis only by microscopic examination, but the final diagnosis should be based on clinicopathological correlation. There is no consensus regarding adjuvant therapy. Both radiotherapy and chemotherapy are recommended in metastatic PTs [2, 6, 16]. Ifosfamide is the most active agent [16]; antiestrogen therapy is not indicated [3, 16]. Some studies revealed several potentially targetable pathway including epidermal growth factor receptor, angiogenesis (vascular endothelial growth factor A, angiopoietin-2, vascular cell adhesion molecule 1, platelet- derived growth factor receptor A, pituitary tumor-transforming1) and immunotherapy (programmed cell death protein 1, programmed death-ligand 1) for patients with locally advanced or metastatic tumors [4, 10]. Park et al. reported a major response to sunitinib and paclitaxel in a case of lung metastatic malignant PT of breast [17].

Little is known about the prognosis of bone metastasis from malignant PT. Nguyen [6] report one case involving the left iliac bone with good response after radiotherapy. The prognosis of malignant PT metastatic to the lung seems to be worse [2].

## Conclusion

In summary, malignant PT is a rare and aggressive fibroepithelial neoplasm. An accurate diagnosis of metastases should be based on clinicopathological correlation allowing exclusion of differential diagnoses. The goal of successful managing this tumor is early detection and complete resection prior to dissemination.

### Consent

Written informed consents were obtained from the patients for publication of these Cases Report and any accompanying images. Copies of the written consents are available for review by the Editor-in-Chief of this journal.

## Abbreviations

PT: Phyllodes tumor
